# Role of Epstein-Barr Virus and Human Papilloma Virus in the Development of Oropharyngeal Cancer: A Literature Review

**DOI:** 10.1155/2022/3191569

**Published:** 2022-06-20

**Authors:** Michela Migliaro, Daniela Massuh, María Fernanda Infante, Ana María Brahm, María Trinidad San Martín, Duniel Ortuño

**Affiliations:** ^1^Escuela de Odontología, Facultad de Medicina, Pontificia Universidad Católica de Chile, Santiago, Chile; ^2^Universidad de Los Andes, Santiago, Chile

## Abstract

The aim of this review was to describe the association and related mechanisms between HPV, EBV, and the development of oral and oropharyngeal cancer. A search for scientific evidence was carried out in electronic databases (MEDLINE/PubMed, SciELO). It was found that, among the carcinogenic mechanisms of HPV, E6 and E7 proteins are responsible for the malignization process, inhibiting tumor suppressors p53 and pRb. As to EBV, it was noted that its “hit and run” phenomenon manipulates the host epigenetic mechanism, triggering the tumor process without the virus being currently present; a “cellular reprogramming” is essentially generated, causing heritable changes in gene expression without DNA mutation. In conclusion, there is an association between oropharyngeal carcinogenesis and HPV and also between the former and EBV. Further studies are required to clarify the causal mechanisms and impact of both viruses on cancer development and to obtain biomarkers of greater specificity in the case of EBV.

## 1. Introduction

Cancer is one of the leading causes of death, and it has become the first or second cause of death before reaching the age of 70 in several countries. According to the World Health Organization (WHO), cancer has led to a sharp decline in mortality rates from heart disease and stroke [1].

Head and neck cancer (HNC) is a worldwide public health problem that includes different types of cancer. HNC constitutes approximately 7% of all existing malignancies, being the fourth leading cause of cancer death in men [2].

Oral cancer (OC) accounts for 2% of all cancers affecting the body, and it is considered the 15th leading cause of cancer mortality in the world [2]. Squamous cell carcinoma (SCC) derives from the stratified squamous epithelium of the oral mucosa, and it is the most common type of oral cancer. In fact, 90–95% of all oral cancers are SCC-type, thus accounting for 5% and 2% of all cancers in men and women, respectively. SCC has a worldwide incidence of 275,000 cases per year, with a survival rate of 50% 5 years after diagnosis, and 20% in later stages [3–5].

Oral cancer is very common in South Central Asia, India, and Melanesia, with Papua New Guinea having the highest incidence rate in both sexes worldwide, which might be associated with betel nut chewing in these countries. Incidence is also high in Eastern Europe, Western Europe, Australia, and New Zealand [1].

Tobacco and alcohol consumption are among the risk factors for SCC and they account for 75% of all cases, while the remaining 25% include infection by Human Papillomavirus (HPV) [6]. It has also been shown that the oral microbiome predisposes to its development. Unbalanced microbiomes and poor oral health can contribute to the process of carcinogenesis in the oral cavity as well as in other areas of the body [6].

In recent studies, it has been reported that approximately 20% of all carcinogenic processes that afflict humans are caused by viruses with oncogenic potential, called oncoviruses. This is due to the chronic inflammation caused by a viral infection, which leads to cell death, cell cycles with uncontrolled proliferation, and modulation of the expression of specific regulatory proteins of the host cell. These oncoviruses manage to decode genes that interfere with the genetic machinery of the infected cells, causing viral replication and the synthesis of viral proteins. The role of viral infection in the tumor process is extensive because it requires the interaction of various factors, such as the compromise of the immune system, certain specific mutations, and exposure to cancerous agents [7]. In recent years, it has been shown that there is an association between the development of oropharyngeal cancer and the oncogenic role of HPV 16. The latter was classified by the International Agency for Research on Cancer (IARC) as a carcinogenic agent for oropharyngeal cancer [8]. Although the relationship between EBV and OSCC has already been established, its carcinogenic role is not completely clear. Further studies are required to fully demonstrate it [9, 10].

The aim of this review was to describe how HPV and EBV are associated with the development of oral and oropharyngeal cancer based on the best available evidence.

## 2. Human Papillomavirus (HPV)

### 2.1. Epidemiology of HPV and Oral Cancer

Two genetically distinct types of head and neck squamous cell cancer (HNSCC) have been determined, the first one is related to alcohol, tobacco, and oral trauma, and the other is associated with HPV infection [2]. The latter is a sexually transmitted oncogenic virus responsible for causing annually about 630,000 cancers worldwide [11]. HPV is a double-stranded DNA virus that infects the stratified flat epithelium of the skin and mucous membranes [4, 12].

HPV infection has been established as an etiologic factor for oral and oropharyngeal cancers [11, 13]. The worldwide prevalence of HPV infection is 45.8% in the oropharynx and 24.2% in the oral cavity [4].

HPV types affecting the mucosa can be divided into high-risk and low-risk groups, with about 15 high-risk HPV types. About 90% of HNSCC are caused by HPV 16 and 18, having a 2.8 times higher probability of causing this type of cancer when compared to other less risky types [14, 15]. HPV 16 and 18 are classified as human carcinogens due to the oncoproteins encoded in their DNA, which can deregulate the cell cycle [4].

Both mortality and incidence of HPV-related HNSCC have increased significantly in recent decades. Concerning the incidence rate, it rose from 16.3% to 71.7% between 1984 and 2004 in the United States, specifically in the case of the oropharyngeal type, which can be related to the increasing incidence of tumor-related HPV [2, 14–17]. According to the SEER (Surveillance Epidemiology and End Results) data, oropharyngeal cancer has shown a significant increase in young adults between the ages of 20 and 44 [18].

Some of the factors that could explain the causal association between HPV and oral cancer are the affinity of HPV for epithelial cells, its oncogenic role, and some morphological similarities between the genital and oropharyngeal epithelium [19].

In addition, oral and oropharyngeal HPV(+) cancers share some characteristics, such as their diagnosis in young people aged under 39 years, high-risk sexual behaviors, and a better prognosis in comparison with their HPV(−) counterparts [11].

### 2.2. Risk Factors for the Development of Oropharyngeal Cancer

#### 2.2.1. Sexual Practices

HPV infection is the most common sexually transmitted infection, and approximately 75% of sexually active adults acquire at least one genital HPV infection in their lifetime [20]. It is uncertain how HPV is transmitted to the oral cavity and causes Oral Squamous Cell Cancer (OSCC). However, sexual behaviors related to the oral cavity could explain HPV infection [14].

Specific sexual practices such as oral sex and oral-anal contact and having multiple sexual partners may favor the occurrence and pathogenesis of HPV in the oral cavity [2, 21]. Furthermore, these practices are significantly associated with the development of cancer in the oropharyngeal region, and this link is stronger in oral HPV-16 infections, independent of tobacco or alcohol consumption [21].

Additionally, there are sites in the oropharynx associated with HPV as they are exposed to the virus, including the base of the tongue, tonsils, soft palate, uvula, and Waldeyer's ring [11]. The increased exposure to HPV that these subsites have may respond to certain sexual behaviors since individuals who engage in high-risk sexual practices are more likely to develop cancer in those areas as reported in the past [11, 21].

#### 2.2.2. Human Immunodeficiency Virus (HIV)

Having multiple sexual partners is also a risk factor for Human Immunodeficiency Virus (HIV) infection. The direct role of HIV in HPV-associated neoplasms is not fully resolved yet. However, HIV infection depresses the immune system against HPV since lower CD4+ lymphocyte levels increase the prevalence of oral, anal, and cervical HPV infection and the incidence of high-grade intraepithelial neoplasia [22].

#### 2.2.3. Tobacco and Alcohol Use

The overall incidence of HNC in the US has decreased in recent years due to lower tobacco use. However, the incidence of HPV-related oropharyngeal cancer appears to be increasing, which is also the case in European countries [23].

Several studies have shown an association between alcohol and tobacco consumption and oral HPV(+) infection in certain types of HNC [2, 11, 13, 14]. Still, a study conducted in Peru suggests that alcohol is not necessarily a direct risk factor for oral HPV infection, especially because of its positive association with HPV(−) oropharyngeal cancer. On the other hand, it was found that those individuals with smoking habits had an almost 3 times higher prevalence of oral HPV infection, regardless of alcohol consumption, suggesting a possible role of smoking in oral HPV infection, which may be caused by a modulation of the immune system [13].

### 2.3. Prevalence by Gender

Several studies have reported that there is a higher prevalence of oral HPV infection and its high-risk types in men as compared to women, in both HPV-related subsites and all anatomical sites studied. This prevalence has increased from 3 to 5 times, being this information consistent with the higher incidence of HNC in the male gender compared to the female. The tendency to affect the male sex could be explained by biological differences or behaviors associated with sexual practices [2, 13]. A 2016 review concluded that within the demographic profile, patients with HPV-related OSCC are mostly male [16]. Likewise, in a study conducted in Brazil, there was a higher incidence in the male gender, in both HPV-related and unrelated sites. However, a study contradicts the above, showing that women younger than 39 years were three times more affected in HPV-related subsites than men (F: 18% versus M: 5.8%) [11].

### 2.4. Mechanism of Oral HPV Infection and Carcinogenesis

HPV infection initiates in the basal stratum of the epithelium, where capsid proteins (L1) come into contact with cell surface receptors. For the HPV to access, it needs a disruption or trauma in the tissue; otherwise, if the epithelium remains undamaged, the virus will be unable to reach its site of action [4]. The oral cavity and oropharynx are ideal sites for infection since they are areas prone to suffer microtrauma during normal physiological activities. Together with orogenital sexual intercourse with carriers, this proclivity makes these regions target sites for repeated attacks by the virus [4].

**Figure 1:**
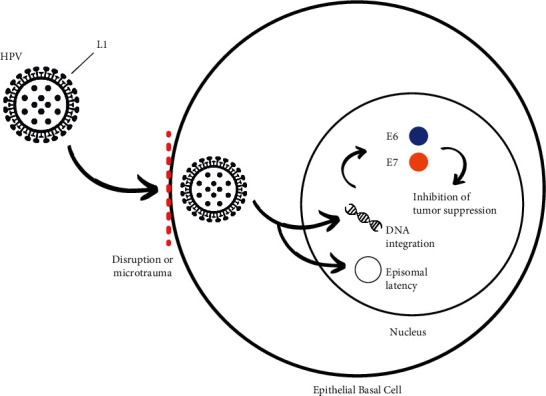
Mechanism of oral infection and carcinogenesis of HPV. In the presence of microtrauma of the basal epithelium of the host cell, HPV enters through interaction between the L1 proteins of its capsid and cell surface receptors. It is then incorporated into the cell nucleus where it can either remain in an episomal state or undergo DNA integration. E6 and E7 proteins inhibit tumor suppression.

Once the virus encounters a target cell, the viral DNA reaches the nucleus, where it can remain in an episomal state or enter a state of active transcription (Figure 1). HPV does not encode proteins that carry out the transcription of its genome; therefore, it depends on the mitosis and replication machinery of the host cell to carry it out. Among the carcinogenic mechanisms used by HPV to achieve cell transformation, the viral proteins E6 and E7 are described as responsible for the malignization process, which will be explained below [4].

One of the mechanisms by which HPV causes cancer is the oncoproteins E6 and E7 of the virus, which inhibit tumor suppressor proteins p53 and retinoblastoma proteins (pRb). In particular, the E6 gene induces the degradation of the p53 protein by interacting with its associated protein (E6AP). On the other hand, E7 binds to pRb and disrupts its binding to genes that encode a group of transcription factors (E2F). Therefore, if E7 inactivates pRb, the latter will not be able to perform its function and prevent the excessive cell growth that takes place in tumors, consequently increasing the susceptibility of the individual to develop cancer. Cell cycle dysregulation caused by HPV infection can lead to the accumulation of DNA damage, thus promoting carcinogenesis [2, 14].

In the mechanism mentioned above, the infected daughter cell abandons the basement membrane where it would leave the cell cycle and cease its DNA replication, but the E6 and E7 proteins play their role, thus preventing the cell from entering a state of genetic nonreplication. Both proteins combine their activities to create a phase of the cell cycle, called pseudo-S, in which the synthesis of genetic material occurs [24]. By inactivating p53, E6 fails to activate p21, which results in a blockage of specific enzymes, causing the release of the transcriptional factor E2F through the phosphorylation of pRb. The latter acts as an oncogene stimulating the transition from G1 to the S phase. It is worth mentioning that under normal conditions pRb should bind to and stop the E2F transcriptional factor [2].

In parallel, E7 binds to pRb through the specific sequence LXCXE, which allows its degradation. E2F is again not properly regulated, favoring the transcription to the synthesis stage of the cell cycle. E7 binds to other pocket proteins that are related to pRb, p107, and p130 as well. This results in increased cellular DNA replication and increased cell division, causing the number of HPV-infected cells to rise [2, 24].

In addition to the mechanism of E6 and E7 oncoproteins, there is a variety of molecular mechanisms for HPV-induced carcinogenesis in the head and neck, including immune evasion, facilitation of genomic instability, virus DNA integration, and changes in global DNA methylation and gene expression [12].

Further studies are, nevertheless, needed to reveal the underlying molecular mechanisms of HPV in oropharyngeal cancer and in turn to develop new preventive and optimal treatment strategies for patients with HPV-related HNSCC [12].

### 2.5. Methods for Detecting HPV

To date, there is no method that is considered the gold standard for HPV detection in the oropharynx. Among the known methods, microbiopsy and brush cytology stand out. The former is a minimally invasive technique that consists of making a cytological smear on the lesion, using a brush or a swab. To be analyzed by a pathologist, the sample collection is placed in a sterile polypropylene cup. Microbiopsy, on the other hand, consists of an excisional process of the lesion, in which the sample obtained is analyzed by a pathologist, along with a second smaller sample subjected to a molecular virological analysis. In the latter, the detection of the virus's DNA is carried out by means of a Polymerase Chain Reaction (PCR), which allows DNA extraction, identification of different HPV genotypes, and data analysis [25].

According to a prospective study, published in 2020, that analyzed these two HPV detection methods and was carried out in sixty-five patients with oral leukoplakia, microbiopsy is more reliable and accurate for detecting HPV and its genotypes than brush cytology. This is in part because it allows access to the most basal layer of the epithelium, where HPV DNA is often located episomally. Despite its advantage, this method is not used as a screening tool since it follows an invasive procedure [25].

Another more recent method was evaluated in a study carried out in 2021. It was based on HPV sequencing (HPV-seq) through the identification of a tumor DNA marker of malignant lesions associated with this virus (ctDNA). The results demonstrated that HPV-seq outperformed digital PCR (dPCR) in detecting ctDNA and by providing quantitative and qualitative information. Therefore, HPV-seq represents a promising and sensitive method for ctDNA detection and analysis in HPV-associated malignancies [26]. As to other detection methods and HPV activity, further studies need to be conducted.

### 2.6. Prognosis of HNSCC according to the Presence of HPV

HPV(+) oropharyngeal SCC has a better prognosis compared with nonvirus-related SCC [2, 16, 27]. For instance, five-year survival rates for patients with an advanced-stage HPV(+) OSCC are 75% to 80%, whereas values for patients with similarly staged HPV(−) tumors are below 50% [2]. The reduction in death could be as high as 60% to 80% in oral and oropharyngeal HPV(+) OSCC [4].

HPV(+) HNSCC has a better long-term prognosis, a lower mutational burden and cell differentiation, and a higher 5-year survival rate. Its overexpression of the p16 biomarker, with functional inactivation of p5, rarely generates metastases and is highly radio- and chemosensitive, as compared to its HPV(−) counterpart (see Table 1) [28, 29].

Furthermore, overexpression of the p16 biomarker is used to detect poorly differentiated and locally more advanced (T4, N2-3) HPV(+) oropharyngeal carcinomas. Therefore, in HPV(+) malignant lesions, an increased expression of this protein could be used as a favorable prognostic marker. On the other hand, loss of p16 expression by deletion, hypermethylation, or mutation is common in alcohol and tobacco-induced OSCC, producing lesions with a worse prognosis as they do not respond in the same way to chemo and radiotherapy [28].

As mentioned above, the HPV E6 oncoprotein can inactivate p53 although in these cases it would be a functional inactivation, not a mutation as it occurs in OSCC associated with tobacco and alcohol intake. In fact, the rate of p53 mutations due to HPV is very low. All this supports the existing evidence that HPV-associated oral and oropharyngeal SCC has longer survival and better prognosis, as compared to its HPV(−) counterpart [28].

### 2.7. HPV Immunization

Clinicians recommend that individuals are vaccinated against HPV, regardless of gender, for the prevention of cancers, such as oropharyngeal cancer [15]. Two vaccines are currently available, the quadrivalent vaccine that protects against low-risk HPV (6 and 11) and high-risk HPV (16 and 18); and the bivalent Cervarix® vaccine, which only protects against high-risk HPV. Both vaccines could be useful in the prevention of high-risk HPV infection and contribute, therefore, to a decrease in the incidence of oral and oropharyngeal cancers [4, 11, 16]. The bivalent vaccine protects against oral HPV infection with an efficacy of 93.3% (95% CI: 63–100%) [14, 16]. Further studies are, however, required to demonstrate the indirect effectiveness of HPV vaccines in the prevention of oropharyngeal cancer.

## 3. Epstein-Barr Virus (EBV)

### 3.1. Epidemiology of EBV and Oral Cancer

Worldwide, the estimated number of people infected with EBV exceeds 5.5 billion [30]. EBV is usually acquired in early childhood, affecting more than 90% of adults [8, 30]. This infection is usually asymptomatic or mild in infants, and in adolescents, it often develops infectious mononucleosis, persisting throughout the individual's life. It is usually misdiagnosed as its symptoms mimic less serious diseases [5, 27, 31].

EBV has been frequently identified in carcinomas located in the oropharyngeal region, whose main risk factors are tobacco and alcohol. However, tumors located at the nasopharyngeal site have been associated with environmental cofactors such as nutritional components, oral hygiene, and host factors. The latter include genetic predisposition, family history, and male gender [30]. An approximately 50% presence of EBV has been reported in cancers located in the upper airways and oral cavity, suggesting that infection with this virus is an important risk factor [27, 32].

### 3.2. Virus Composition and Routes of Transmission

**Figure 2:**
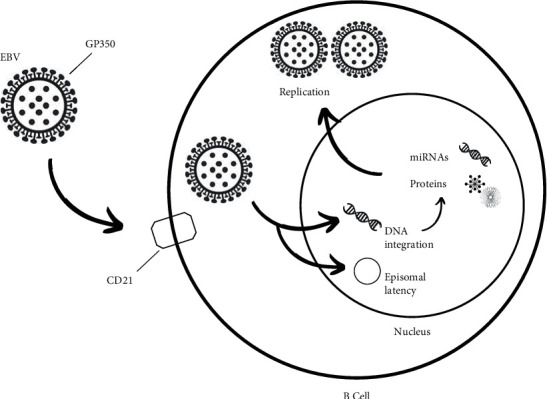
Mechanism of EBV infection: EBV accesses the B lymphocyte, where it can enter a replicative cycle producing virions or it can remain as a latent infection, persisting throughout the individual's life.

EBV belongs to the oncogenic human herpesvirus family, and it is composed of linear double-stranded DNA enveloped in a capsid [31]. The route of entry of the virus is through the viral Gp350 glycoproteins present in its envelope, which bind to the CD21 receptors of B lymphocytes. Another route is through virgin epithelial cells, by endocytosis or by fusion with their plasma membrane [5, 27]. Once inside the cell, in addition to remaining as an extrachromosomal episome, the virus continues to replicate by forming new virions (Figure 2). It presents itself in a biphasic form in its viral cycle, which is very convenient for its long-term persistence. Thus, its latency in memory B lymphocytes or epithelial cells causes viral gene expression to be silenced, preventing its elimination by cytotoxic T lymphocytes. Consequently, once the host is infected, it develops a lifelong infection [27].

The oral cavity is the primary site of infection and reservoir of the virus, mainly in the tonsils and Waldeyer's ring. Replication occurs in the upper differentiated layers of epithelial cells, and when the virus is transferred to the saliva, it becomes a form of transmission, with even asymptomatic patients being carriers of the virus. Additionally, the virus can be transmitted through transplants and transfusions [5, 27].

### 3.3. Associations between EBV and Neoplastic Processes

Although the virus was originally isolated in Burkitt's lymphoma, recent findings have clarified its oncogenic role, due to its association with various neoplastic processes. Among these are Hodgkin's lymphoma, non-Hodgkin's lymphoma, natural killer T-cell lymphoma, nasopharyngeal carcinoma (NPC), gastric carcinoma, and SCC [5, 27, 31]. EBV genes have been shown to activate oncogenes such as Bcl-2 and MYC, which trigger different signaling pathways and inhibit tumor suppressor genes such as p53. This provides a survival advantage for the host cell to evade the host's immunogenic responses [31, 33]. Still, further evidence on the mechanisms by which the virus participates in the development of oral cancer is required [9, 27].

In all malignant lesions, the virus is in a latent phase, a state that evades the host immune system while allowing sustained expression of viral oncogenes [27]. In addition, high viral loads have been correlated with tumor progression, as seen in various types of malignant lesions [27, 32]. To enter this latency state, the EBV uses six nuclear antigens (EBNA1, EBNA2, EBNA3A/B/C, EBNA-LP) and three latent membrane proteins (LMP1, LMP2A/B). There is strong evidence showing that some of these EBV proteins are associated with oncogenicity [33].

The EBV genome can exist as episomes or extrachromosomal copies of the genetic material. These express latent genes during different stages of B-cell differentiation in vivo. In the case of EBV, it expresses the latency III program or one of the two alternative forms of virus latency (known as latency I and latency II), expressing different products depending on its latency state (Table 2).

### 3.4. Impact of EBV on Epigenetics

In recent years, a phenomenon has been observed in which EBV generates an epigenetic “cellular reprogramming” in lymphoid and epithelial cells. In these cells, heritable changes in gene expression occur, not involving a mutation in the DNA sequence itself. This malfunction of epigenetic machinery can have a direct impact on the expression of tumor suppressor genes and oncogenes, which could lead to tumor generation. Some of these epigenetic alterations associated with tumor generation include DNA methylation, histone modification, and chromatin conformation alterations. In fact, the impact of EBV on epigenetic regulation is evident and it takes part in the development of different cancers; for example, EBNA1, which is the protein that maintains the EBV episome, is detectable in every type of EBV-associated neoplastic process. It can bind to some promoter regions of oncogenes and tumor suppressor genes, regulating their expression in B lymphocytes and epithelial cells [33].

**Figure 3:**
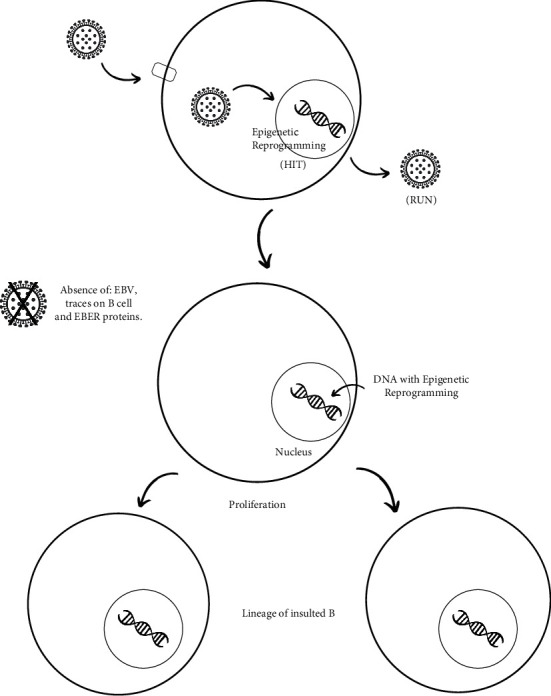
EBV enters the B lymphocyte, causing a series of changes in DNA expression. Following the insult, epigenetic changes and silencing of genes expressing viral antigens occur in the host lymphocyte. Subsequently, EBV disappears completely from the insulted cell, and a process of B-cell proliferation begins in the same cell. In this manner, the daughter cells inherit the DNA reprogrammed by EBV, without the virus being present in the proliferation cycle.

It has been shown that malignancies involving EBV have altered states of DNA methylation in tissues that are EBV(−). Specific viral factors manage to manipulate the epigenetic mechanism of the host, triggering the progression of the tumor process; it does not necessarily require the current presence of the virus for this to occur [27]. This mechanism has been called “hit and run” and it works as follows: when EBV infects a B lymphocyte, it delivers the information that requires to grow and survive, and it makes it potentially oncogenic, which is known as “hit.” When this occurs, a competent immune system eliminates these infected B lymphocytes because it recognizes the EBV viral antigens on their surface. This is precisely what the virus has learned to prevent from occurring as it induces epigenetic changes and silencing of the genes that express the viral antigens in the host lymphocyte, known as “run.” Such changes become heritable in the cell lines, even when the virus is no longer present, thus becoming the modus operandi to produce neoplastic development (Figure 3). As it has been seen, in the successor cell lines of infected cells, the selection of B lymphocytes that show less viral dependence for survival and growth begins to be favored, confirming the stated above [34].

Concerning the cell cycles of EBV(+) cells, in the first cycle, about 8% of the viral genome is lost, and after eight cycles, 50% of its DNA has been lost. After fifty cycles, only 1% of the viral genetic information remains in the cell lines, being undetectable by conventional methods, such as the use of the EBER biomarker [35]. This was observed in a study that analyzed six Burkitt's lymphoma samples, in which EBER markers were used and all were determined to be EBV(−). However, after performing the virus coding microRNA detection method, all six samples were shown to have this marker and, therefore, the EBV viral genome. This would support the theory of the hit-and-run mechanism and could be a very sensitive tool for the early diagnosis of neoplastic processes, even in cases categorized as EBV(−) [35]. These findings could explain other types of cancers, which do not necessarily present high levels of the viral genome to be associated with a virus that is generating or contributing to the development of a neoplastic process.

Several primary studies and systematic reviews have established EBV as a risk factor for OSCC. Also, it is described that the location of EBV is common in the tonsils and base of the tongue, sites considered EBV reservoirs [5]. However, within the literature, epidemiological studies range from those excluding any relationship to those that recognize a strong association between EBV and OSCC. This may be due to different factors, such as geographic and ethnic differences, in addition to the use of methods and biomarkers with low sensitivity and specificity that have been used to detect EBV [5, 27].

Over the years, studies have recognized an association between EBV infection and HNC. Despite this, the evidence on the incidence of EBV-derived cancers is contradictory [27]. For instance, some studies have demonstrated high levels of the virus in tumors, thus considering these EBV(+) tumors; while, in other studies, tumors were considered EBV(−) as no virus genome was found in them. In the latter, however, the neoplastic process could have been initiated by the virus through the “hit-and-run” mechanism, where EBV could have unleashed the neoplastic process and “escaped,” without leaving its viral genome in the tumor [27].

These disparate results may be explained by the role of the virus in tumor development and progression mainly. The emergence of new markers has clarified this contradiction in the studies, recognizing the role of EBV in the development of the neoplastic process even though its genome is no longer present [31].

Among the markers for detecting the presence of the virus are the genes of viral proteins involved in cell proliferation, immune response, and apoptosis. These genes regulate these processes and produce cellular immortalization, which is important for the appearance of neoplasia [31]. Noncoding RNAs called “EBER” were long used as markers to detect EBV [27]. However, it was found that many samples that were EBER (−) did have EBV present in the tumor, resulting in a sensitivity of about 67.8% for this marker [27, 35, 36].

Lately, new methods in molecular biology that are much more sensitive and specific have been developed and are being studied. In neoplastic cells of Burkitt's lymphoma EBER(−), the presence of microRNA coding for the virus has been demonstrated. However, studies are still needed to implement these tools, which would clarify the role of the virus in the neoplastic process, the loss of viral genomes in some tumors studied, and the difficulty of analyzing the spontaneous occurrence of cancers. Finally, the use of coding microRNA markers could be the missing piece in the “hit-and-run” theory of tumor progression [35].

## 4. EBV/HPV Coinfection and Association with Oral Cancer

It is estimated that globally between 15% and 20% of carcinomas affecting the oropharynx are coinfected with EBV and HPV. However, in the case of SCC in the tonsils and base of the tongue, coinfection rates have been seen to vary from 25% to 70% [27].

One study showed that EBV/HPV coinfection increases the replication of both viruses when cultured together in vitro. During coinfection, HPV may alter the viral cycle of EBV, favoring its latency. On the other hand, EBV delays epithelial differentiation and promotes an invasive phenotype, further enhancing the invasiveness of epithelial cells expressing the E6 and E7 oncogenes of HPV-16 [27].

In a study published in 2020, EBV and high-risk HPV coinfection was analyzed in HNSCC tissues from Bosnian patients. A 34.7% coinfection rate was observed in the samples, being strongly associated with advanced-stage HNSCC (*p* value 0.03). This suggests that both viruses interact in the progression of these cancers [37].

Although evidence is still lacking to define how EBV might contribute to the pathogenesis of HPV(+) oropharyngeal cancers, this could be explained by the influence of HPV and EBV on each other's viral cycles [27].

## 5. Conclusion

There is an association between oropharyngeal carcinogenesis, HPV, and EBV, independently and also when both of the viruses are present. The main carcinogenic mechanism of HPV is through E6 and E7 proteins, which inhibit tumor suppressors p53 and pRb. As to EBV, the tumor process is triggered without the virus necessarily being present; this phenomenon was called “hit and run.”

Further studies are required to clarify the causal mechanisms and contribution of both viruses to cancer development. Regarding HPV, future research should focus on analyzing the effectiveness of new preventive strategies, including HPV immunization for oral and oropharyngeal cancer. In the case of EBV, the introduction of new detection techniques and biomarkers of greater specificity is needed.

## Figures and Tables

**Table 1:** Prognostic difference between HPV(+) and HPV(−) HNSCC.

	HPV(+)	HPV(−)
Mutational load	Lower	Higher
Cell differentiation	Low	Marked
5-year survival rate	75–80%	<50%
Biomarkers	Overexpression of P16, inactivation of p53	Loss of P16, p53 mutation
Metastasis	Infrequent	Frequent
Radiosensitive/chemosensitive	+++	+

**Table 2:** Expression products in each of the latency states of EBV in EBV-associated neoplasms. Viral genes and small RNAs are observed in the different types of EBV latency that can be seen in the different neoplasms.

Diseases	EBNA-1	EBNA-2 A/B	EBNA-3 A/B/C/LP	LMP 1	LMP 2	EBERs	EBV latent type
Burkitt lymphoma (BL)	+	−	−	−	−	+	I
Nasopharyngeal carcinoma (NPC)	+	−	−	+	+	+	II
Hodgkin lymphoma (HL)	+	−	−	+	+	+	II
NK/T-cell lymphoma	+	−	−	+	+	+	II
Diffuse large B-cell lymphoma	+	+	+	+	+	+	III
